# Peri-operative factors predisposing to pharyngocutaneous fistula after total laryngectomy: analysis of a large multi-institutional patient cohort

**DOI:** 10.1186/s40463-017-0233-z

**Published:** 2017-08-23

**Authors:** Nicole L. Lebo, Lisa Caulley, Hussain Alsaffar, Martin J. Corsten, Stephanie Johnson-Obaseki

**Affiliations:** 1 Department of Otolaryngology - Head and Neck Surgery, University of Ottawa, S3, 501 Smyth Road, Ottawa, ON K1H 8L6 Canada; 2 Department of Otolaryngology, Aurora Health Care, Aurora St. Luke’s Hospital, Milwaukee, WI USA

**Keywords:** Laryngectomy, Pharyngocutaneous fistula, Predisposing factors, National surgical quality improvement program, Peri-operative

## Abstract

**Background:**

Pharyngocutaneous fistula (PCF) is a problematic complication following total laryngectomy. Disagreement remains regarding predisposing factors. This study examines perioperative factors predicting PCF following total laryngectomy using a large multicenter data registry.

**Methods:**

Retrospective cohort analysis was performed using patients undergoing total laryngectomy in the ACS-NSQIP database for 2006–2014. Sub-analysis was performed based on reconstruction type. Outcome of interest was PCF development within 30 days.

**Results:**

Multivariate analysis of 971 patients was performed. Three variables showed statistical significance in predicting PCF: wound classification of 3 and 4 vs. 1–2 (OR 6.42 *P* < 0.0004 and OR 8.87, *P* < 0.0042), pre-operative transfusion of > 4 units of packed red blood cells (OR 6.28, *P* = 0.043), and free flap versus no flap reconstruction (OR 2.81, *P* = 0.008).

**Conclusions:**

This study identifies important risk factors for development of PCF following total laryngectomy in a large, multi-institutional cohort of patients, thereby identifying a subset of patients at increased risk.

## Background

Pharyngocutaneous fistula (PCF) is a common, problematic, and frustrating complication following total laryngectomy (TL) – a procedure central to the management of many laryngeal cancers. PCF is associated with longer hospital stays, delays in adjuvant therapy, discomfort, and quality of life loss for patients [1]. Rates of this complication have often been quoted as anywhere from 3 to 65% [2]; a recent meta-analysis suggests rates are within the 10–25% range [3].

Significant disagreement exists regarding predisposing factors for PCF. Most studies investigating risk factors have been relatively small and single center [4–6], which have produced conflicting results. To date, no large, multi-institutional studies using prospectively-gathered data have been published.

Three systematic reviews assessing risk factors for PCF post TL have been published [1, 2, 7]. The most consistently identified risk factors have been a post-operative hemoglobin of < 125 g/L, and a history of radiotherapy. Other factors that have been identified in at least one of the systematic reviews include prior tracheostomy, concurrent neck dissection, tumor subsite (supraglottis), positive surgical margins, advanced primary tumor (T3-T4), history of chronic obstructive pulmonary disease (COPD), receipt of blood transfusion, previous combined chemoradiotherapy, hypopharyngeal involvement, and use of catgut suture during closure [1, 2, 7].

The objective of this study is to bridge the current literature gap by examining perioperative factors predicting PCF development following TL using data from a large, multi-institutional registry. The ultimate aim of this study is to assist in guiding operative planning and perioperative optimization for TL patients.

## Methods

### Study design

A retrospective cohort analysis was performed using data from the American College of Surgeons – National Surgical Quality Improvement Program (ACS-NSQIP) database for the years 2006 to 2014. NSQIP is a registry of prospectively-gathered demographic, comorbid, and perioperative variables collected for patients undergoing non-cardiac procedures at participating centers worldwide. The ACS-NSQIP database currently draws data from over 750 hospitals, primarily in the USA, Canada, Australia, and Middle Eastern countries [8]. Data is collected by rigorously-trained nurse reviewers, and has been well-validated [9]. Patients are routinely tracked for 30-days post-operatively. NSQIP data is de-identified, and available to all institutions complying with the ACS-NSQIP Data Use Agreement.

### Population

**Fig. 1:**
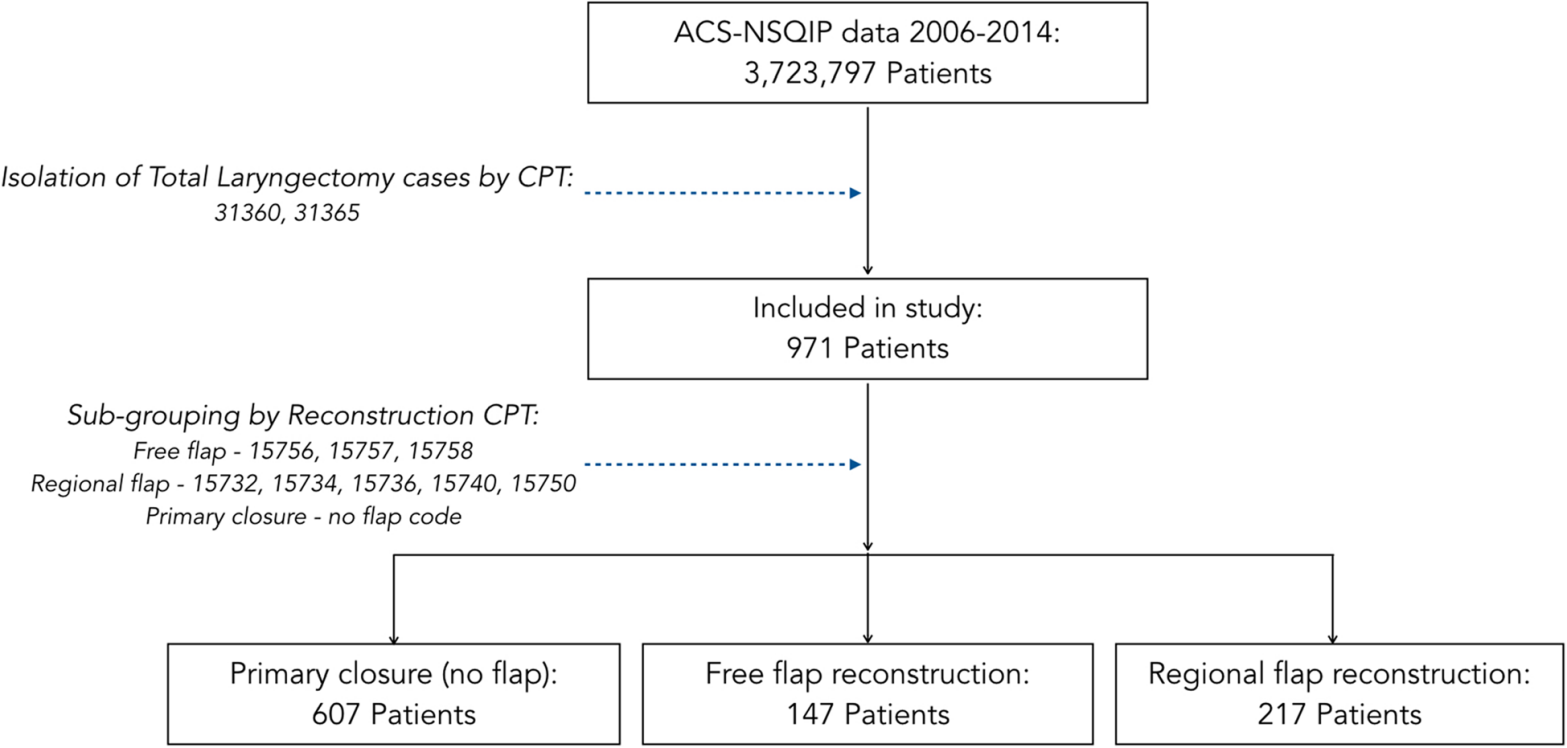
Patient Selection Algorithm. Legend: ACS-NSQIP = American College of Surgeons - National Surgical Quality Improvement Program database; CPT = Current Procedural Terminology code

The study population of interest – patients in the NSQIP database undergoing total laryngectomy (primary or salvage) – was isolated using Current Procedural Terminology (CPT) codes. Total laryngectomy procedures were categorized by one of two CPT codes: 31,360 and 31,365 – total laryngectomy without or with radical neck dissection, respectively. Patients undergoing partial or subtotal laryngectomy, and patients undergoing total pharyngectomy with total laryngectomy were excluded from analysis (see Table 1 for summary of relevant CPT codes). Using secondary CPT codes, patients were further divided into three sub-groups based on reconstruction type: patients were classified as having undergone (1) primary closure (i.e., no flap reconstruction), (2) free flap reconstruction, or (3) regional flap reconstruction (classification algorithm summarized in Fig. 1).

**Table 1:** Relevant Current Procedural Terminology (CPT) Code Definitions

CPT Code	Definition
Primary procedure	
31,360	Laryngectomy; total, without radical neck dissection
31,365	Laryngectomy; total, with radical neck dissection
31,367	Laryngectomy; subtotal supraglottic, without radical neck dissection
31,368	Laryngectomy; subtotal supraglottic, with radical neck dissection
31,370	Partial laryngectomy (hemilaryngectomy); horizontal
31,375	Partial laryngectomy (hemilaryngectomy); laterovertical
31,380	Partial laryngectomy (hemilaryngectomy); anterovertical
31,382	Partial laryngectomy (hemilaryngectomy); antero-latero-vertical
31,390	Pharyngolaryngectomy, with radical neck dissection; without reconstruction
31,395	Pharyngolaryngectomy, with radical neck dissection; with reconstruction
Flap procedure	
15,732	Muscle, myocutaneous, or fasciocutaneous flap; head and neck (eg, temporalis, masseter muscle, sternocleidomastoid, levator scapulae)
15,734	Muscle, myocutaneous, or fasciocutaneous flap; trunk
15,736	Muscle, myocutaneous, or fasciocutaneous flap; upper extremity
15,740	Flap; island pedicle requiring identification and dissection of an anatomically named axial vessel
15,750	Flap; neurovascular pedicle
15,756	Free muscle or myocutaneous flap with microvascular anastomosis
15,757	Free skin flap with microvascular anastomosis
15,758	Free fascial flap with microvascular anastomosis
Other	
31,611	Construction of tracheoesophageal fistula and subsequent insertion of an alaryngeal speech prosthesis (eg, voice button, Blom-Singer prosthesis)

*CPT* Current Procedural Terminology

### Outcome and variables

The primary outcome of interest was development of PCF within 30-days following TL. In the absence of procedure-specific variables, the NSQIP variable “wound-breakdown” was used as a proxy for PCF, recognizing that PCF will represent the majority of these occurrences, but acknowledging that this may capture patients with superficial skin dehiscence as well. The variables analyzed for prediction of PCF were: age, gender, body mass index (BMI), perioperative comorbidities (diabetes, smoking, COPD, congestive heart failure, bleeding disorder, hypertension), functional status, pre-operative wound infection, chronic steroid use, weight loss, perioperative blood transfusion, American Society of Anesthesia (ASA) classification, wound classification, concomitant tracheoesophageal puncture (TEP) insertion, and type of reconstruction (free flap, regional flap, primary closure). All variables were treated in either binary or categorical fashion (see Table 5 in Results section for detailed NSQIP variable definitions). Of note, the ACS-NSQIP database defines perioperative transfusions into 3 comparison groups: pre-operative transfusion of more than 4 units of pRBCs within 48 h of the operation; intra-operative pRBCs transfused in the operating suite; and postoperative transfusion of more than 4 units of PRBCs within 72 h of the operation. Wound classification was treated as a categorical variable (that is, 1–2, 3 and 4) where sufficient sample size permitted. If insufficient sample size resulted in model instability, the classifications were analyzed as a binomial variable. The variables in the regression analysis were selected because they had either been identified as risk factors for PCF in previous literature, were demographically important, or were relevant to clinical judgment.

### Statistical methods

Univariate followed by multivariate logistic regression analysis assessing odds ratios for the above variables were performed for the entire cohort and within groups stratified by reconstruction type. All statistical analysis was performed using SAS 9.3 software (SAS Institute Inc., Cary, NC, USA), with significance defined as *P* < 0.05.

## Results

Of the 3,723,897 patients within the ACS-NSQIP database, 971 were identified to meet the study criteria of having undergone a total laryngectomy, with or without radical neck dissection. Of these patients, 607 (62.5%) were closed primarily, 147 (15.1%) were reconstructed using a free flap, and 217 (22.3%) were reconstructed using a regional flap. Patient demographics for the reconstructive groups as well as the population as a whole are detailed in Table 2. Age distribution of patients was fairly consistent between all reconstruction groups, with the mean age of all patients being 62.8 years (SD 11.4). Male-female ratio varied somewhat between groups, with the regional flap group having the highest male-predominance (M:F 4.95:1), and the free flap group having the lowest (M:F 2.97:1).

**Table 2:** Study patient demographics – overall and by reconstruction-type sub-grouping

	All patients	1° closure	Free flap	Regional flap
N	971	607	147	217
Gender				
Male	776	483	110	183
Female	195	124	37	37
M:F ratio	3.98	3.90	2.97	4.95
Age (years)				
Mean (std dev)	62.8 (11.4)	63.1 (12.0)	61.6 (10.3)	62.8 (10.7)
Maximum	90	90	83	86
Minimum	20	20	37	31

*Std dev* standard deviation, *M:F* male to female ratio

Of the 971 total patients, 50 developed PCF, for a rate of 5.1%. Within the subgroups, rates were 3.8% (23/607) for primary closure, 9.5% (14/147) for free-flap reconstruction, and 5.5% (12/217) for regional flap reconstruction. Rates summarized in Table 3.

**Table 3:** Rate of pharyngocutaneous fistula development in all patients and by reconstruction type

Group	Rate of PCF development
Overall (all patients)	5.1%
Primary closure	3.8%
Free flap	9.5%
Regional flap	5.5%

*PCF* Pharyngocutaneous fistula

For the overall group (all patients), a univariate followed by multivariate analysis was performed with each of the variables listed in the methods section; the multivariate results are displayed in Table 4. Three factors were identified through multivariate analysis to be statistically significant predictors of PCF development in the overall group. Wound class – contaminated and dirty (3 and 4) compared to clean/clean-contaminated (1–2) – had the strongest correlation, with an odds ratio of 6.42 (95% CI 2.30–39.45, *P* = 0.0004) and 8.87 (95% CI 1.99–39.45, *P* = 0.004), respectively. The other factors were: transfusion of more than 4 units of packed red blood cells (PRBCs) within 72 h pre-operatively (OR 6.28, 95% CI 1.06–37.30, *P* = 0.04), and reconstruction with a free flap compared to primary closure (OR 2.81, 95% CI 1.31–5.99, *P* = 0.008). A decreased incidence of PCF in patients undergoing regional flaps compared to those with free flaps was suggested but did not reach statistical significance (OR 0.51, 95% CI 0.21–1.24, *P* = 0.14)

**Table 4:** Multivariate regression analysis of risk factors for PCF in all patients

Variable	Comparison	Odds Ratio	95% CI	*P*-value
Age	Increase by 10 years	1.05	0.78–1.42	0.75
Gender	Male vs. female	0.66	0.32–1.36	0.26
BMI	≥ 18.5 vs. < 18.5 kg/m^2^ (underweight)	0.67	0.30–1.51	0.33
Diabetes	Yes vs. no (Identified DM on oral agents or insulin in 30 days pre-op)	1.55	0.64–3.73	0.33
Smoking	Yes vs. no (current smoker within 1 year)	1.48	0.76–2.89	0.24
Functional Status	Dependent vs. independent (in last 30 days)	0.84	0.27–2.63	0.76
COPD history	Yes vs. no (history severe COPD)	0.72	0.31–1.67	0.44
CHF history	Yes vs. no (within last 30 days)	1.33	0.14–12. 43	0.80
HTN requiring medication	Yes vs. no	0.81	0.41–1.57	0.52
Pre-op wound infection	Yes vs. no (open or infected wound at site at time of OR)	1.83	0.50–6.71	0.36
Chronic steroid use	Yes vs. no	2.39	0.80–7.20	0.12
Weight loss	> 10% loss in 6mo pre-op vs. < 10%	0.91	0.40–2.06	0.82
Bleeding disorder history	Yes vs. no identified history	1.52	0.31–7.54	0.61
Pre-op transfusion	> 4 units PRBCs vs. ≤ 4 (in 72 h pre-op)	6.28	1.06–37.30	0.04
Wound class	Contaminated vs. Clean/Clean-contaminated	6.28	2.29–17.94	0.0004
	Dirty vs. Clean/Clean-contaminated	8.87	1.99–39.45	0.004
ASA Class	3–5 vs. 1–2	0.93	0.30–2.82	0.89
Peri-op or post-op transfusion	Yes vs. no (any transfusion of PRBC or whole blood from start of OR to 72 h post-op)	0.80	0.09–6.85	0.84
TEP insertion during procedure	Yes vs. no	1.13	0.51–2.50	0.77
Reconstruction type	Free flap vs. Primary closure	2.81	1.31–5.99	0.008
	Regional flap vs. Primary closure	1.45	0.67–3.11	0.34
	Regional flap vs. Free flap	0.51	0.21–1.24	0.14

*PCF* Pharyngocutaneous fistula, *CI* confidence interval, *BMI* body mass index, *DM* diabetes mellitus, *COPD* chronic obstructive pulmonary disease, *CHF* congestive heart failure, *HTN* hypertension, *PRBCs* packed red blood cells, *ASA* American Society of Anesthesiologists, *TEP* tracheoesophageal puncture

For the primary closure group, univariate followed by multivariate analysis was again performed using the same variables. Only variables identified in the univariate analysis as being statistically significant (*P* < 0.05) were included in the multivariate analysis. The results of the multivariate analysis are displayed in Table 5. One statistically significant risk factor was identified in this group: BMI ≥ 18.5 compared to < 18.5 (underweight) with an odds ratio of 0.28 (95% CI 0.11–0.73, *P* = 0.009) – meaning a normal or greater BMI was correlated with lower PCF rates. Of note, one variable (peri/pre-op transfusion) was omitted from the analysis for the primary closure group because of low event numbers causing model instability.

**Table 5:** Multivariate regression analysis of risk factors for PCF in patients receiving primary closure

Variable	Comparison	Odds Ratio	95% CI	*P*-value
Age	Increase by 10 years	0.99	0.68–1.43	0.95
Gender	Male vs. female	0.94	0.33–2.70	0.91
BMI	≥ 18.5 vs. < 18.5 kg/m^2^ (underweight)	0.28	0.11–0.73	0.009
Pre-op wound infection	Yes vs. no	5.12	0.88–29.63	0.068
Chronic steroid use	Yes vs. no	3.28	0.89–12.13	0.07
Wound class	Contaminated vs. Clean/Clean-contaminated	3.01	0.56–16.16	0.20
	Dirty vs Clean/Clean-contaminated	2.50	0.24–26.38	0.44

*PCF* Pharyngocutaneous fistula, *CI* confidence interval, *BMI* body mass index

For the free flap reconstruction group, univariate analysis showed no statistically significant risk factors – possibly due to insufficient numbers (only 147 patients in this group) – thus multivariate analysis was not performed. Of note, univariate analysis could not be performed on multiple variables (functional status, history of CHF, ASA class, and peri−/post-op transfusion) because of low event numbers causing model instability.

For the regional flap reconstruction group, univariate analysis showed only one statistically significant risk factor: wound class – contaminated/dirty (3–4) compared to clean/clean-contaminated (1–2) – with OR 17.6 (95% CI 4.57–67.71, *P* < 0.0001). Wound class could not be analyzed as a categorical variable for this subgroup due to small sample sizes in the contaminated and dirty cohorts. Multivariate analysis was not performed. Of note, again for this group, multiple variables had to be omitted from univariate analysis because of insufficient event numbers (history of CHF, wound infection/open wound at time of operation, chronic steroid use, bleeding disorder, and pre-op transfusion).

## Discussion

For head and neck surgeons and patients alike, PCF is a frustrating complication following total laryngectomy. Much effort has been expended by single institutions to investigate rates and predisposing factors for PCF [4–6] to guide potential preventative measures and identify those at high risk for such a complication. Our goal was to fill the current literature gap using a large, prospectively-gathered, multi-institutional database to study PCF risk factors, with the goal of improving understanding, and providing higher quality evidence via access to a larger study population.

Of the 917 patients included in this study, 50 (5.1%) developed PCF. This falls within the classically quoted 3–65% range in literature [2]. It is, however, lower than rates identified in recent single-institution studies, which have been in the 30–35% range [4–6]. Given the wide range of PCF rates identified between centers, it is particularly advantageous here to access and analyze a large database like NSQIP with rigorous and consistent data collection to help dilute what appears to be an institution/surgeon-dependent influence on PCF rate.

With respect to identified risk factors for PCF in all-comers, our analysis determined three factors to be of statistical significance. The first of these was wound class (contaminated or dirty, compared to clean or clean-contaminated). We observed an incremental increase in odds of PCF with progression from contaminated to dirty wound classification (OR 6.42 vs 8.87, respectively), which lends further strength to this finding. Wound classification was not a factor identified in any of the three systematic reviews [1, 2, 7] as predisposing to PCF, although it is clinically intuitive as a risk factor for wound breakdown secondary to infection. Wound infection has been identified as a risk factor in a single institution study previously [10]. In addition, more advanced wound class may also reflect tumor involvement of the skin causing an open wound at the time of OR – a finding consistent with advanced tumor stage. Advanced tumor stage has been identified in numerous studies as an independent risk factor for PCF development [7, 11, 12]. In the same vein, existing tracheostomy at time of OR could also explain an advanced wound classification; prior tracheostomy has also been found in a number of studies to be a predictor of PCF [2, 4, 13]. Surgeons should exercise extreme vigilance in monitoring these patients in the post-operative setting given their high risk status for PCF. Neither tumor stage nor prior tracheostomy were captured in the NSQIP data, thus these factors were not assessed independently in this study. Of note, pre-operative wound infection was not found to be significant in the univariate regression analysis in our study. The reason for this finding is likely two-fold: 1) small sample size, and 2) surgeon selection bias. In our study, only 30 of 971 patients were identified with wound infection. As such, it is possible that the parameter did not reach significance in our regression model due to lack of statistical power in the cohort analysis. In addition, there is likely a component of surgeon selection bias as these patients would be treated preoperatively and only few patients would present to the OR with active wound infection. Future studies with larger sample sizes would be necessary to offer greater sensitivity for this particular covariate.

The second risk factor for PCF identified in the overall group was transfusion of more than 4 units of PRBCs within 72 h pre-operatively. This is consistent with systematic review findings that transfusion and anemia (HGB < 125 g/L) are significant risk factors [1, 2, 7]. Of note, while pre-operative transfusion was correlated with PCF in our study, peri- and post-operative transfusion were not found to be significantly so. This likely reflects different indication for transfusion in these two time periods; pre-operative transfusion is likely triggered by pre-operative anemia, which often reflects chronic disease and poor nutritional status, which are established risks for poor wound healing [14]. Peri- and post-operative transfusions, on the other hand, are likely triggered by intraoperative blood loss, which is unlikely to be related to the patient’s nutritional status. These findings would emphasize the need for pre-operative management of nutritional and immune status in surgical patients. With optimization of surgical patients and close post-operative monitoring, it is possible that the rates of post-operative complications due to this subset of patients may decline.

The third factor identified in our total population was reconstruction with a free flap compared to primary closure. This is an interesting finding given previous literature suggesting that use of free flaps is protective against wound breakdown compared to wounds closed primarily [3, 4, 15]. We suspect this aberrant finding is secondary to confounding effects of a disproportionate number of patients undergoing free flap closure having had previous radiation compared to the primary closure group, since a history of local radiation is often the indication for free-tissue transfer. Previous radiotherapy has been fairly consistently identified as a risk factor for PCF in the literature [1, 2, 7, 16], however, radiation exposure is not well captured in the NSQIP database, and thus this factor could not be controlled for (NSQIP only records radiation therapy received within 30 days prior to operation; salvage laryngectomy usually occurs more than 30 days after primary radiotherapy). These findings should prompt further consideration of primary closure of the neopharynx where surgically feasible in total laryngectomies. Further studies are needed to better determine the risk of wound breakdown in previously radiated patients. Regardless, surgical patients who do require free flap reconstruction should be closely monitored for signs of PCF in the post-operative setting.

We then divided our analysis of factors predicting PCF by reconstruction-type: primary closure, regional flap closure and free tissue transfer (free flap). This particular stratified analysis was performed to control for selection bias introduced by surgeon choice in reconstruction-type. Beginning with the primary closure group, multivariate analysis identified being underweight (BMI < 18.5) at time of OR was identified as an effect modifier for PCF in this subgroup of patients. Interestingly, BMI is a unique risk factor not identified in the overall group. The correlation of low BMI with increased rates of PCF is consistent with the finding of several studies that have identified pre-operative albumin (as a proxy for pre-operative nutritional status) as a risk factor for PCF [17–19]. This is also consistent with our earlier discussion supposing pre-operative anemia predisposes to PCF because it reflects poor pre-operative nutritional status. These findings would again support the need for pre-operative optimization of nutritional and immune status and vigilant monitoring in the post-operative setting for patients with these identified risk factors. Of note, the PCF rate for the primary closure group was quite low, at 3.8%. This may be the result of bias towards primary laryngectomy instead of salvage laryngectomy in this group, as a primary (i.e., non-radiated) field is more amenable to primary closure. Accordingly, similar to the comparison between closure types, incomplete capture of previous radiation may be influencing these results as well. Multivariate analysis could not be performed for the other two subgroups. In the regional flap group, only one statistically significant risk factor was identified with univariate analysis: wound class – contaminated/dirty (3–4) compared to clean/clean-contaminated (1–2). Low numbers precluded us from performing a multivariate analysis in this group. Similarly, in the free flap group, multivariate analysis could not be performed.

While NSQIP is a useful tool due to its size and multi-institutional nature that allows for generalizability for results, there are limitations in its use, as there are with all databases. The key limitations of this study are: (1) confounding effects from variables not captured by NSQIP, (2) low event numbers once patients divided into subgroups, (3) selection bias from surgeon selection of reconstruction type, and (4) information bias from reliance on retrospective data. With respect to the first limitation – NSQIP is a general surgical database, thus data is not captured with laryngectomy and its complications specifically in mind. As we are accessing the data retrospectively, we are limited to what is currently available in the database, which unfortunately does not capture a number of variables that are suspected to influence laryngectomy outcomes, such as: previous tracheostomy, previous radiotherapy or chemotherapy (beyond 30 days pre-operatively), tumor staging, tumor subsite, and surgical margins. Accordingly, these variables could not be controlled for in our analysis and further studies are needed to establish the impact of these variables in the rates of PCF development. As such, the risk factors that we identified in our study may not be an exhaustive list of potential predictors of post-operative PCF. With respect to the second limitation – despite the size of the NSQIP database, once divided into groups for further analysis based on reconstruction type, low event numbers within the groups limit meaningful analysis. Particularly in the free flap and regional flap groups, where either one or no variables were identified as statistically significant risk factors on univariate analysis, risk of type II error was clearly higher than in the overall group. Only within the primary closure group, with 607 patients, were we able to perform a meaningful multivariate analysis.

An important limitation to recognize in this particular study is the bias introduced by the use of “wound-breakdown” as a proxy for PCF, in the absence of targeted documentation of PCF occurrence. As previously discussed, PCF will represent the majority of these “wound-breakdowns” and thus is a reasonable parameter to measure for this research question. However, there is a risk of positive bias due to the fact that our measured rate may represent an overestimate of PCF rate amongst our population. One would expect patients with only superficial incisional dehiscence to be included in this catchment, thereby artificially inflating the measured rate. As well, this parameter has the potential to introduce negative bias if the measured rate underestimates the risk of PCF due to misclassification of PCF by surgeons or data entry technicians. However, the rate of PCF was found to be 5.1%, which falls within the classically quoted 3–65% range in literature [2]. This allowed for reassurance that this parameter was still able to represent the outcome of interest with measured accuracy.

Despite these limitations, our study does derive strength from the high quality of the NSQIP database, which draws information from hundreds of centers to provide large samples sizes, has rigorous data collection methods with trained data collectors, and is prospectively gathered. In addition, the fact that the database draws from many centers worldwide improves the generalizability of our results.

## Conclusion

In summary, this is the largest multi-center study evaluating the risk factors for PCF using prospectively-gathered data to date. Identified statistically significant risk factors of PCF for all-comers were: wound class, pre-operative transfusion, and free-flap reconstruction compared to primary closure. These factors should prompt surgeons to consider close monitoring in the post-operative setting for PCF, given the higher risk in these selected patients. As the NSQIP data set continues to evolve, particularly to include more patients and procedure-targeted data, more in-depth and nuanced analysis will become possible.

## Abbreviations

ACS-NSQIP: American college of surgeons national surgical quality improvement program
ASA: American society of anaesthesia
BMI: Body mass index
CHF: Congestive heart failure
CI: Confidence interval
COPD: Chronic obstructive pulmonary disease
CPT: Current procedural terminology
DM: Diabetes mellitus
HGB: Hemoglobin
HTN: Hypertension
PCF: Pharyngocutaneous fistula
PRBCs: Packed red blood cells
TEP: Tracheoesophageal puncture
TL: Total laryngectomy
